# Mortality incidence and its determinants after fragility hip fractures: a prospective cohort study from an Egyptian level one trauma center

**DOI:** 10.4314/ahs.v21i2.41

**Published:** 2021-06

**Authors:** Mohammad K Abdelnasser, Ahmed A Khalifa, Khaled G Amir, Mohammad A Hassan, Amr A Eisa, Wael Y El-Adly, Ahmed K Ibrahim, Osama A Farouk, Hossam A Abubeih

**Affiliations:** 1 Orthopedic Department, Assiut University Hospital, Assiut, Egypt; 2 Orthopedic Department, Qena faculty of medicine and its University Hospital, South valley university, Qena, Egypt; 3 Public Health and Community Medicine Department, faculty of medicine, Assiut University, Assiut, Egypt

**Keywords:** Fragility hip fractures, trochanteric fractures, mortality rate

## Abstract

**Background:**

Fragility hip fracture is a common condition with serious consequences. Most outcomes data come from Western and Asian populations. There are few data from African and Middle Eastern countries.

**Objective:**

The primary objective was to describe mortality rates after fragility hip fracture in a Level-1 trauma centre in Egypt. The secondary objective was to study the causes of re-admissions, complications, and mortality.

**Methods:**

A prospective cohort study of 301 patients, aged > 65 years, with fragility hip fractures. Data collected included sociodemographic, co-morbidities, timing of admission, and intraoperative,ostoperative, and post-discharge data as mortality, complications, hospital stay, reoperation, and re-admission. Cox regression analysis was conducted to investigate factors associated with 1-year mortality.

**Results:**

In-hospital mortality was 8.3% (25 patients) which increased to 52.8% (159 patients) after one year; 58.5% of the deaths occurred in the first 3-months. One-year mortality was independently associated with increasing age, ASA 3–4, cardiac or hepatic co-morbidities, trochanteric fractures, total hospital stay, and postoperative ifection and metal failure.

**Conclusion:**

Our in-hospital mortality rate resembles developed countries reports, reflecting good initial geriatric healthcare. However, our 3- and 12-months mortality rates are unexpectedly high. The implementation of orthogeriatric care after discharge is mandatory to decrease mortality rates.

## Introduction

A fragility fracture is defined by the World Health Organization as “a fracture caused by an injury that would be insufficient to fracture a normal bone as a result of reduced compressive and/or torsional strength of bone”^1^. Fragility hip fracture is considered a rising worldwide healthcare problem^2^. In 2000, the reported worldwide incidence of hip fractures in people aged >50 years was approximately 1.6 million^3^. With aging and expansion of the world population, the annual estimate of fragility hip fractures is expected to reach 2.6 million by 2025 and 4.5 million by 2050 4. In the Middle East, approximately 52,000 hip fractures were recorded in 1990, which is suspected to increase to 192,000 by 2025 and to 435,000 by 2050 5.

A recent systematic review by Downey C et al. in 2019, included data from 8 national hip fracture registries and studies reporting one-year mortality covering 36 countries, they found that the mean one-year mortality rate was 22% (ranging from 2.4% to 34.8%) 6. The highest risk of mortality occurs within three months 7, 8; however, the mortality remains high compared to the agematched controls for as long as ten years^9^.

Apart from increasing mortality, a high percentage of physical and mental morbidities with increasing disability, loss of independence, and increased level of institutionalization may follow^10^–^12^. This explains the high amount of health and socioeconomic burdens posed by this problem^13^, ^14^.

Most of the literature analysing mortality and morbidity after fragility hip fractures come from developed countries; little information comes from the Middle East and from low and middle income countries (developing countries)^15^. Moreover, there are many controversies about the risk factors predicting mortality associated with fragility hip fractures. To the best of our knowledge there was no detailed mortality rate report after fragility hip fractures from our area (Africa and the Middle East) in the past five years. To help us with proper implementation of a geriatric care program attacking the most significant factors affecting mortality at a proper time, we carried the current study.

## Aim

The primary objective of this study was to evaluate the mortality rate (in-hospital, 3-months, 6-months, and one year) after the management of fragility hip fractures in an Egyptian population. The secondary objective was to study the causes of complications, re-admissions, and mortality.

## Methods

We conducted a prospective cohort study for all patients diagnosed with a fragility hip fracture admitted to the trauma unit in our institution (level 1 Trauma Centre) from January 2016 to December 2016. Patients less than 65 years old, periprosthetic fractures, and pathological fractures were excluded. Informed consent was obtained from all the patients or their caregivers before enrolling the subjects for this study. The ethical committee of our institution approved the study (IRB no.: 17100171).

### Pathway of patients with fragility hip fracture

As the patient with suspected fragility hip fracture arrives at the emergency department at our hospital (in the current series 75.5% of patients presented at the same day of trauma, 24.5% presented within one week after trauma),

### Evaluation

The first evaluation and history taking are performed by an orthopaedic resident including details of trauma mechanism, preinjury activity level, and preexisting medical comorbidities. Full physical examination (general and local) is performed. Prescribing appropriate analgesia before transferringthe patient to the radiology department, usually, an AP pelvis and a lateral view of the injured hip are performed. After confirming the diagnosis, non-adhesive skin traction is applied to the injured limb (in case of trochanteric fractures).

### Admission

the patient is admitted and transferred to a standard inpatient trauma ward, and anticoagulation in the form of low molecular weight heparin should be initiated unless contraindicated. Preparation of the patient for surgery is initiated within 8 hours after admission after consultation of internist and anesthesiologist (when needed). If the patient is ready for surgery (from a medical and surgical perspective), it is performed within 36 hours after admission (anticoagulation is stopped 8 hours before surgery).

### Surgery

Patients were given priority in the operative list, and choice of anesthesia is according to the preference of the anesthesiologist (either neuraxial or general). All surgeries were performed by well-trained orthopaedic surgeons (at least two years of experience dealing with such cases). Surgical decision and device to be used were according to the policy of our department (for trochanteric fractures patients, fixation was performed using a sliding hip screw, and for patients with neck of femur fracture, all received a cemented bipolar hemiarthroplasty).

### Post-operative

patients were transferred to the recovery area for at least 8 hours; critical patients were transferred to the ICU. Postoperative plain radiographs were obtained, then patients were transferred to the ward, the usual medications prescribed postoperatively are antibiotics, analgesics, and anticoagulants (started 12 hours postoperative). Full blood picture is performed the first day postoperatively, and blood transfusion was advised if the Hb level is below 8 g/dl. Patients having hemiarthroplasty were allowed for an assisted toe-touch weight-bearing protocol at postoperative day one.

### Rehabilitation

Weight-bearing was restricted for Patients with trochanteric fractures; however, mobilization in bed at least once each day was done with assistance from members of the health care staff, including nurses. Where safe and appropriate, family members or caregivers were encouraged to assist with daily mobilization.

### Discharge

Since there was no specialized orthogeriatric care unit, Patients usually were discharged from the hospital by postoperative day three unless they had either a medical or a surgical complication necessitating their stay at the hospital. Patients were either discharged to their home or the nearest health facility if needed. Patients were transferred to the nearest hospital (if needed).

### Follow up

Follow up visits were scheduled at two weeks for suture removal, six weeks for radiographs recheck, three months, six months, 12 months, and then annually. Patients were advised to visit the hospital if any major incident happened between these intervals, or at least make a telephone call for any inquiries. In case of death, the relative or the caregiver was asked about the time and place of death and whether the patient was admitted to any hospital before death or not.

### Data collection

Two independent researchers collected the data via a structured questionnaire designed specifically for this study that contains demographic data (age, sex, residence, smoking, co-morbidities, American Society of Anaesthesiologists (ASA) score, type of the fracture, the timing of the trauma before hospital admission and causes of delay if any), intraoperative data (type of operation, timing after admission and causes of delay if any, and intraoperative complications or mortality), postoperative in-hospital data (length of stay, complications, mortality), and post-discharge data which were collected at 3, 6, and 12 months (complications, mortality, re-admission). The STROBE guidelines were used to ensure the quality of reporting of this observational study^16^.

### Statistical analysis

Data were analysed using SPSS version 21* (IBM-SPSS Inc, Chicago, IL, USA). Frequency tables were examined to explore missing data, errors in the data, and data inconsistency. Missing data were treated by replacing the missing value with median values. Descriptive statistics such as means, standard deviations, medians, and percentages were calculated. The Chi-square test or Fisher's Exact test was used to compare the difference in the distribution of frequencies among different groups. For continuous variables, independent t-test analysis and one-way ANOVA were carried out to compare the means of normally distributed data, while the Mann-Whitney U test and Kruskal-Wallis test were calculated to test the median differences of the data that do not follow a normal distribution. The relationships between patient characteristics and survival were analysed by the Kaplan-Meier and Cox Regression Analyses (Forward LR). Age and sex were added as priori variables, and the clinical and demographic factors with proven statistical significance from the univariate analyses were further included in the multivariate Cox Hazard Regression models. A P-value of ≤ 0.05 was regarded as significant.

## Results

During the study period, 362 patients with fragility hip fractures were admitted to the Trauma Unit of our Hospital. Three pathological fractures and four periprosthetic fractures were excluded. We lost 14.9% (54 patients) to follow-up after discharge. This left 301 patients eligible for this study. The basic characteristics of the patients are demonstrated in (Table 1).

**Table 1:** Basic characteristics of the studied patients

Variable	Category	n = 301(100%)
**Age in years** (Mean ± SD)	74.2 ± 0.47	
**ASA Class.**	ASA (1 & 2)	n = 254 (84.4%)
	ASA (3 & 4)	n = 47 (15.6%)
**Sex**	Male	n = 151 (51.2%)
	Female	n = 150 (49.8%)
**Residence** 1	Rural	n = 166 (55.1%)
	Urban	n = 135 (44.9%)
**Co-morbidity** (DM, HTN, Cardiac, hepatic disease)	Yes	n = 111 (36.9%)
	No	n = 190 (63.1%)
**Cause of Trauma**	Fall on ground	n = 278 (92.4%)
	Others (Road Traffic Accidents, Fall from Height)	n = 23 (7.6%)
**Diagnosis**	Trochanteric fracture	n = 172 (57.1%)
	NOF fracture	n = 112 (37.2%)
	Others (Subtrochanteric, and acetabular fractures)	n = 17 (5.7%)

ASA; American Society of Anesthesiology, DM; Diabetes mellitus, HTN; hypertension, NOF; neck of the femur

1 Rural refers to patients who reside in villages at the periphery of the city where our trauma center is located (about 40 km far). In contrast, Urban refers to patients living within the city.

**Figure 1:**
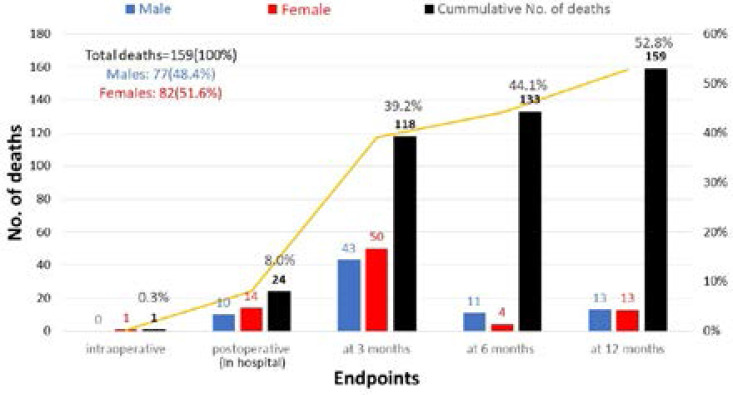
Mortality rate at each study endpoints (stratified by sex).

Regarding the mortality rate, in-hospital mortality (one patient died intraoperatively and 24 postoperative) was 8.3 % (25 patients), 3-month mortality was 39.2 % (118 patients), 6-months mortality was 44.1 % (133 patients), and at 12 months follow-up a total of 159 patients died constituting a one-year mortality rate of 52.8 %, of those deaths, 48.4% (77 patients) were males, and 51.6% (82 patients) were females, the overall survival after 1-year was 47.2% (142 patie/span>nts) (Figure 1).

Complications occurred in 19.3% (58 patients), which was distributed as follows: intra-operative blood loss that necessitated blood transfusion occurred in 3% (9 patients), chest infection (pneumonia) in 1% (3 patients), and revision of fixation in 0.3% (1 patient). Deterioration of the general condition with ICU admission occurred in 1% (3 patients). After discharge, surgical site infection occurred in 11.3% (34 patients), and metal failure in 3% (9 patients).

Re-admission was required in 7.3% (22 patients). The most common reason for re-admissions was infection in 36.3% (8 patients), metal failure in 27.3% (6 patients), non-surgical causes in 27.3% (6 patients), and unrelated operations in 9.1% (2 patients). Attrition rates were found as follows: In-hospital attrition rate 8.6%, at 3 months 40%, at 6 months 8.5% and after completing 1 year it was 16.8%.

Factors associated with 1-year mortality and significance of each was calculated by running a univariate analysis as shown in (Table 2).

**Table 2:** Univariate analysis for factors potentially associated with one-year mortality

		Alive (No=142)	Dead (No=159)	P-value
Age in years (Mean ± SD)		71.9 ± 6.9	76.2 ± 8.6	< 0.001*
Sex	Male	73 (48.7%)	77 (51.3%)	= 0.344†
	Female	69 (45.7%)	82 (54.3%)	
Residence	Rural	82 (49.4%)	85 (50.5%)	= 0.392†
	Urban	60 (44.5%)	75 (55.6%)	
**Co-morbidity**	DM	20 (37%)	34 (63%)	= 0.055†
	HTN	12 (38.7%)	19 (61.3%)	= 0.192†
	Cardiac	5 (29.4%)	12 (70.6%)	= 0.041†
	Hepatic	0 (0%)	19 (100%)	< 0.001†
**Diagnosis (n=287)**	NOC fracture	67 (59.3%)	46 (40.7%)	= 0.002†
	Trochanteric fracture	71 (40.8%)	103 (59.2%)	
**ASA Classification**	ASA (1 & 2)	129 (50.8%)	125 (49.2%)	= 0.004†
	ASA (3 & 4)	13 (27.7%)	34 (72.3%)	
**Complications after** **operation**	No	134 (57.8%)	98 (42.2%)	< 0.001†
	Yes	8 (11.6%)	61 (88.4%)	
**Re-admission**	No	134 (57.8%)	98 (42.2%)	< 0.001†
	Yes	8 (11.6%)	61 (88.4%)	
**Timing of** **Operation**	< 48 hours	68 (51.9%)	63 (48.1%)	= 0.282†
	48 – 96 hours	37 (46.3%)	43 (53.7%)	
	> 96 hours	37 (41.1%)	53 (58.9%)	
**Hospital Stay in days** (Mean ± SD)		5.65 ± 2.5	6.86 ± 3.6	= 0.003‡

DM; Diabetes mellitus, HTN; hypertension, NOF; neck of femur, ASA; American Society of Anesthesiology.

* independent t-test was used to compare the mean difference between the two groups

† Chi-square analysis was used to compare the difference in proportions

‡ Mann Whitney U test to compare the median difference between the two groups

-- Significance level is considered when p ≤ 0.05

Identifying factors as a risk for 1-year mortality after hip fractures were done using the multivariate Cox Hazard regression analysis as shown in (Table 3). The following factors were identified as the risk factors for 1-year mortality after hip fractures: age, ASA 3–4, trochanteric fractures, associated cardiac disease, associated hepatic disease, total hospital stay, and postoperative morbidity (infection, and metal failure).

**Table 3:** Multivariate analysis for risk factors for one-year mortality

		HR^1^ (95% CI)	Adjusted HR^2^ (95% CI)	LRT^3^ P-value
**Age in years (Increase of 1 year)**		1.04 (1.02–1.06)	1.04 (1.03–1.08)	< 0.001
**Sex (Female/Male)**		1.08 (0.79–1.47)	1.12 (0.81–1.56)	= 0.502
**ASA (3&4 vs. 1&2)**		1.75 (1.14–2.69)	1.82 (1.19–2.77)	= 0.006
**Surgical Diagnosis**	NOF fracture	1	1	
	Trochanteric fracture	1.51 (1.07–2.14)	1.64 (1.15–2.33)	= 0.006
**Co-morbidity**	Non- Cardiac Disease	1	1	
	**Cardiac** **Disease**	1.49 (0.80–2.68)	1.68 (1.11–2.95)	= 0.041
	Non- Hepatic disease	1	1	
	**Hepatic Disease**	2.02 (1.37–3.24)	2.13 (1.17–3.61)	<0.001
**Timing of** **Operation**	< 48 hours	1	1	
	48 – 96 hours	1.48 (0.89–2.45)	1.39 (0.83–2.32)	= 0.211
	> 96 hours	1.23 (0.73–2.07)	1.19 (0.69–2.04)	= 0.520
**Total Hospital Stay in** **days (Increase of 1 day)**		1.06 (1.01–1.62)	1.08 (1.03–2.13)	= 0.01
**Post-operative** **Morbidity**	No	1	1	
	Infection	3.31 (2.03–5.07)	4.56 (2.56–6.61)	<0.001
	Metal Failure	1.89 (1.07–3.91)	1.85 (1.04–3.16)	= 0.036
**Re-admission**		1.60 (0.94–2.72)	1.36 (0.79–2.33)	= 0.266

1 HR=Hazard Ratio

2 Adjusted HR=Mutually adjusted CI= Confidence Interval

3 LRT=Likelihood Ratio Test.

ASA; American Society of Anesthesiology. NOF; neck of femur.

## Discussion

Fragility hip fractures considered as a significant public health concern mostly associated with increased morbidity as well as mortality compared to other osteoporos related fractures^17^, ^18^, prolonged recumbency related complications mainly deep vein thrombosis, pulmonary embolism, and pneumonia, which may occur during hospitalization or even after patients discharge had been considered as the leading causes for increased mortality rates^18^–^20^.

In our cohort, we found an in hospital mortality (8.3%) similar to previous studies, 159 died by the end of 1-year constituting a total mortality rate of 52.8% of the whole study population; most of these mortalities were reported at 3-months follow up which represented 58.5% of the total mortalities reported in our study. We found that age, advanced ASA grade (3 or 4), associated cardiac or hepatic disease, trochanteric fractures, post-operative infection or metal failure, and length of hospital stay were significantly associated with mortality.

Hue et al. conducted a meta-analysis that included 75 studies involving 64,316 patients and reported that the overall inpatient or 1-month mortality was 13.3%, 3–6onths was 15.8%, one-year 24.5%, and after 2-year it reached up to 34.5% 21.

We reported 8.3% of in-hospital mortality which resembles what had been reported from developed countries, for example, the stated rates were between 1.6% and 1.8% in USA^22^, ^23^, 5.4% in Italy^24^, 6.3% in Canada^25^, 15% in UK^26^, and 1.3% in Turkey^27^.

In our study, 58.5% of the total deaths occurred in the first 3-months, which resembles what was reported by some authors. Holvik et al. reported 58% of the total mortalities to happen in the first 3-months in their study of 567 patients with fragility hip fractures^28^. Lopez et al. reported a higher frequency of mortality 65.3% during the first 3 months after fragility hip fractures, which then plateaued^8^.

Our 1-year mortality rate was 52.8%, which was as high as what was historically reported from the USA by Beals RK. who showed a 50% mortality rate for patients with hip fractures admitted between 1956 and 1961 29, however, recently the mortality rates have decreased as low as 21% in USA^30^, 8.1% in Italy31, 11.5% in Japan^32^, 23% in Netherlands^33^, 23.5% in Norway^28^, 24.8% in Sweden^34^, 33.5% in the UK^35^, and 22.5% in Spain^8^, this decrement may be attributed to the advancements in fracture stabilization techniques, orthogeriatric care, and increased awareness about healthcare problem associated with fragility hip fractures.

Mortality reports at 1-year from developing countries also showed variable rates, in Thailand, it was reported to be 18%^36^, 30% and 35% in Brazil^37^,^38^, Tunisia, Saudi Arabia and Sudan (representing an African and Middle East countries) the rates were 28.4% 15, 26.98%^39^ and 16.7% 40 respectively.

In concordance with most authors^8^, ^15^, ^27^, ^33^–^38^, ^41^–^43^, we also observed that increasing age is a risk factor for mortality. However, Holvih et al. could not find a correlation between age and 1-year mortality in a study consisting of 567 patients with hip fracture and aged above 65 years^28^. A similar finding was also reported by Mossey et al. in a group of 219 patients with hip fractures^44^. Different cut-off values were reported for increasing mortality: >70 years43, >80 years8, and > 85 years^45^.

We did not detect gender as a risk factor for mortality after hip fracture, which was in agreement with many other studies^27^, ^28^, ^41^, ^42^. However, the effect of gender on mortality after hip fracture is debatable. Male gender has been reported by many authors to be a risk factor for increased mortality after hip fracture^8^, ^15^, ^33^–^38^, ^43^, ^46^, ^47^.

Lopez et al. found that the risk of death was 2.44-folds higher in males8. Endo et al. observed more complications and higher mortality in males during the postoperative hospital stay; at 1-year post-operation, the risk of death for males were double than that of females^46^. A similar finding was also reported by Carpintero et al. who suggested that men have a poor nutritional status and more co-morbidities compared to women; this, in turn, increases the likelihood of death after sustaining a hip fracture^48^. Wehren et al. suggested that infections, such as pneumonia and septicaemia, are more common in male patients, a finding that could explain the higher mortality in male patients^49^. On the contrary, Otzuruk et al. found that the female gender is a risk factor for mortality due to the frailty of females in their population^50^.

As reported in most of the literature, associated co-morbidities increase the risk of death^41^, ^43^, ^51^. The patients with ASA 3 and 4 are at the highest risk^27^, ^28^, ^39^. Regarding the type of co-morbidity, we found that the riskiest co-morbidities were hepatic, followed by cardiovascular co-morbidities.

Ercin et al. identified central nervous system co-morbidities as a specific condition that affects mortality^27^, whereas Sepah et al. stated that cyclic vomiting syndrome co-morbidities are the most dangerous15. Some studies^15^, ^27^ reported increased mortality with >2 different co-morbidities in the same patient. Roche et al.^35^ stated that >3 co-morbidities are the most significant preoperative risk factor, especially respiratory diseases, and malignancy.

Unlike many authors who could not find a correlation between the fracture type and mortality rates^8^, ^15^, ^27^, ^38^, ^42^, in our study, mortality was significantly higher in cases with extracapsular fractures. Similarly, Keene et al. observed higher mortality and morbidity in the extracapsular fracture group^26^.

Early surgery, within 24 hours, maybe challenging to achieve, especially in a medically unfit patient who needs more time for general condition optimization^27^,^50^, ^52^. However, it is generally agreed that hip fractures should be stabilized as early as possible, as recommended by the Royal College of Physicians^52^. Weil et al. reported that in Israel by 2019, more than 85% of hip fracture patients received early surgery (within 48 hours after admission), this led to a reduction of the national 1-year mortality of less than 19%^53^.

In our study, we did not observe a significant association between early surgery and reduced mortality. This is in contrast to the findings of Colais et al., who reported lower 1-year mortality in patients with hip fractures operated within two days of admission^54^. Bottle et al.^55^, as well as Elliott et al.^56^, reported the same findings. On the other hand, other studies failed to find a correlation between early surgery and mortality^28^, ^34^, ^42^, ^50^, ^57^

The most common causes for readmission in our study were infection (36.3%), followed by medical causes (27.3%). Hyes et al. found that the most common reason for re-admission after fragility hip fractures was medical complications, especially bronchopneumonia^58^. The strengths of this study include a large number of patients treated in the same center by a dedicated team which is expected to employ a uniform standard of care and, hence, provide more reliable results. However, this study had several limitations: firstly, as our hospital is a level-1 trauma center with a huge catchment area of more than 20 million inhabitants; therefore, after patients being discharged from our center, different postoperative rehabilitation protocols were applied in various centers, which mostly lacked the concept of orthogeriatric specialized care, which may have its effect on increased mortality rates which we considered as a major limitation of this study. Secondly, as the substantially high number of patients (14.9%) lost to follow up and received their postoperative care and rehabilitation in other hospitals, they were included only in in-hospital mortality and were excluded from the remaining univariate and multivariate analyses. Thirdly, patients included in the study are treated in a trauma service that offers care free of charge, these patients mostly had a low socioeconomic status, and lacked proper care at home after hospital discharge; and possibly if the hip fracture patients with a higher socioeconomic state were included with better home care, this would have changed the mortality rates. Lastly, we compared the results from the current study with what had been reported in the western populations which may have different demographic characteristics that affect the outcome, even the ethnicity of the study group can affect the study outcomes as reported in a study by Lakstein et al.^59^, the main reason behind this is the paucity of detailed published reports during the last 5 years on mortality or morbidity rates after fragility hip fracture from our part of the world (Africa or the Middle East).alized due to financial and logistic reasons however, we are in the process of implementing this program to be part of the standard of care.

Further multicenter studies including national as well as nearby countries trauma institutions should be initiated to define the morbidity and mortality incidence among fragility hip fracture patients in our locality and its possible determinants.

Establishment of an African hip registry to deal with all issues related to fragility hip fractures and its economic burden is mandatory.

## Conclusion

Our in-hospital mortality rate was close to what had been reported from developed countries, reflecting good standards of initial geriatric care provided in the study setting. However, 3- and 12-months mortalities were unexpectedly high, reflecting the deficiency in the socioeconomic aspect of fragility hip fractures care. We believe that lack of rehabilitation centres, deficiency of proper geriatric postoperative care programs and economic reasons are the main factors for the high mortality rate.
